# Are Nonhuman Primates Good Models for SARS?

**DOI:** 10.1371/journal.pmed.0030411

**Published:** 2006-09-26

**Authors:** Robert J Hogan

I would like to respond to Haagmans and Osterhaus' Perspective [1] on Lawler et al.’s study [2]. I have been actively studying SARS virus both in vitro and in numerous animal models including mice, cotton rats, ferrets, and macaques since April, 2003 (about six weeks after the virus was first identified). While I do concur with some of the statements made by Haagmans and Osterhaus, I am compelled to provide an alternative view. I completely agree with the stance that an animal model which mimics the severe disease observed in human cases is needed. However, the continued use of nonhuman primates in these studies is simply not warranted. Indeed, multiple groups have tried unsuccessfully to reproduce this model, including Lawler and colleagues [2] . For example, I attended the WHO meeting on SARS in Rotterdam in February, 2004, at which Steven Jones, of the National Microbiology Laboratory, Winnipeg, Canada, said: “If I were one of those monkeys, maybe I’d just take a Tylenol” [3].

With my colleagues, I conducted a study in which both rhesus and cynomolgus macaques were infected with SARS-CoV. I did not see any clinical signs of disease or marked lung pathology [4]. A study by Subbarao and colleagues had similar findings: “SARS coronavirus (SARS-CoV) administered intranasally and intratracheally to rhesus, cynomolgus and African Green monkeys (AGM) replicated in the respiratory tract but did not induce illness” [5].

Perhaps the most interesting issue is that Lawler and colleagues clearly state that “SARS-CoV infection of cynomolgus macaques did not reproduce the severe illness seen in the majority of adult human cases of SARS” [2]. To my knowledge, only Osterhaus’s laboratory and laboratories from China have reported severe disease in SARS-CoV infected macaques. Osterhaus mentions that the variability in results may be due to factors such as the strain of virus used, and this is certainly true. However, he has not released the virus isolate used in these studies to me or my colleagues in spite of requests. Given that so many groups (e.g., the Centers for Disease Control and Prevention, the United States Army Medical Research Institute of Infectious Diseases, the National Institute of Allergy and Infectious Diseases, etc.) with excellent scientific skills and credentials have reported contradictory results with at least two strains of SARS-CoV, it is troublesome that the use of nonhuman primates in SARS pathogenesis, vaccine, and therapeutic testing continues.
